# Arginine in the FARM and SARM: A Role in Chain-Length Determination for Arginine in the Aspartate-Rich Motifs of Isoprenyl Diphosphate Synthases from *Mycobacterium tuberculosis* †

**DOI:** 10.3390/molecules23102546

**Published:** 2018-10-06

**Authors:** Raimund Nagel, Jill A. Thomas, Faith A. Adekunle, Francis M. Mann, Reuben J. Peters

**Affiliations:** 1 Roy J. Carver Department of Biochemistry, Biophysics and Molecular Biology, Iowa State University, Ames, IA 50011, USA; rnagel@iastate.edu (R.N.); thomas.jillann@gmail.com (J.A.T.); 2 Department of Chemistry, University of Wisconsin-Parkside, Kenosha, WI 53141, USA; adeku001@rangers.uwp.edu

**Keywords:** terpene, isoprenoid, divalent metal co-factor ligation

## Abstract

Isoprenyl chains are found in many important metabolites. These are derived from precursors of the appropriate length produced by isoprenyl diphosphate synthases (IDSs). The human pathogen *Mycobacterium tuberculosis* makes various isoprenoids/terpenoids, with important roles in their biosynthesis played by two closely related IDSs, encoded by *grcC1* (Rv0562) and *grcC2* (Rv0989c), with Rv0989c generating the 10-carbon precursor (*E*)-geranyl diphosphate (GPP), and Rv0562 the 20-carbon precursor (*E,E,E*)-geranylgeranyl diphosphate (GGPP). Intriguingly, while Rv0562 contains the prototypical *trans*-IDS first and second aspartate-rich (DDxxD) motifs (FARM and SARM, respectively), Rv0989c uniquely contains arginine in place of the second Asp in the FARM and first Asp in the SARM. Here site-directed mutagenesis of the corresponding residues in both Rv0562 and Rv0989c reveals that these play a role in determination of product chain length. Specifically, substitution of Asp for the Arg in the FARM and SARM of Rv0989c leads to increased production of the longer 15-carbon farnesyl diphosphate (FPP), while substitution of Arg for the corresponding Asp in Rv0562 leads to increased release of shorter products, both FPP and GPP. Accordingly, while the primary role of the FARM and SARM is known to be chelation of the divalent magnesium ion co-factors that assist substrate binding and catalysis, the Arg substitutions found in Rv0989c seem to provide a novel means by which product chain length is moderated, at least in these *M. tuberculosis* IDSs.

## 1. Introduction

Isoprenoids play essential roles in many metabolic pathways. In large part, due to the hydrophobic nature of polyprenyl chains, these are used to anchor proteins to the membrane or, after cyclization, form the sterols or hopanoids that are integral membrane molecules. Polyprenol-derived molecules such as dolichol are essential in the transfer of glycosyl chains from the cytoplasmatic face of the Endoplasmatic Reticulum (ER) to the ER-lumen where they are attached to proteins, while others such as quinones are involved in electron transport chains. In addition, short-chain isoprenoids (≤20-carbons) can be formed into natural products, termed terpenoids, which directly exert various biological activities [1,2,3]. 

**Figure 1:**
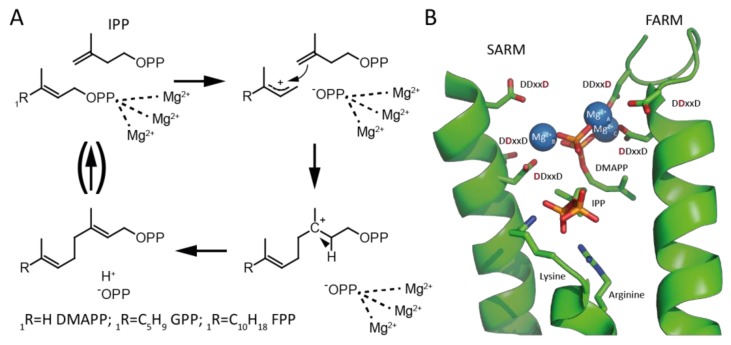
*Trans*-isoprenyl diphosphate synthase (IDS) reaction mechanism and coordination of substrate diphosphate moieties in the active site of *trans*-IDS. (**A**) The allylic substrate is coordinated by three Mg^2+^ co-factors that assist initiating ionization of the diphosphate ester. The resulting carbocation is attacked by the double bond of isopentenyl diphosphate (IPP), leading to formation of a bond between the allylic substrate and IPP and a shift of the carbocation. The resulting carbocation is quenched by deprotonation and formation of a new *trans*/(*E*)- double bond in the elongated product. This product can act as the allylic substrate for further elongation with another IPP molecule. (**B**) The active site of the (*E,E*)-farnesyl diphosphate synthase from *E. coli* complexed with its substrates IPP and dimethylallyl diphosphate (DMAPP) (Protein Data Bank (PDB) ID code: 1RIQ). The diphosphate moiety of the allylic DMAPP is activated by coordination to three Mg^2+^ that are, in turn, bound by the characteristic first and second aspartate-rich DDxxD motifs (FARM and SARM), as shown. More specifically, Mg^2+^_A_ and Mg^2+^_C_ are coordinated by the first and last aspartate of the FARM, while Mg^2+^_B_ is largely coordinated by the first aspartate of the SARM. By contrast, the diphosphate moiety of IPP is more directly bound by the basic residues shown. Note that, while the structure contains the thiolo analog of DMAPP, for illustrative purposes the color of the sulfur was changed to red to resemble that of the oxygen found in DMAPP.

Polymerization of the appropriate length chains from the universal precursors isopentenyl diphosphate (IPP) and dimethylallyl diphosphate (DMAPP) is catalyzed by isoprenyl diphosphate synthases (IDSs). IDS can be divided on the basis of the double bond configuration, either *trans*/(*E*)- or *cis*/(*Z*)-, formed in their prenyl diphosphate products [4]. Shorter chain length prenyl diphosphates are generally produced by *trans*-IDS, although there are a few exceptions [5,6,7,8]. These short prenyl diphosphates with *trans* carbon-carbon double bonds can then be the allylic substrates for *cis*-IDS that catalyze further polymerization with IPP and produce long chain prenyl diphosphates such as decaprenyl diphosphate or undecaprenyl diphosphate, in which the newly added carbon–carbon double bonds are then in the *cis* conformation. Although the *trans*- and *cis*- IDSs both catalyze similar reactions, utilizing divalent metal cation co-factors/substrates (usually magnesium, Mg^2+^) to initiate ionization of the diphosphate ester bond in their allylic substrate (e.g., DMAPP) that then condenses with the terminal alkene of IPP followed by proton elimination [9,10], these form two structurally distinct enzymatic families [4]. For example, while the *cis*-IDS utilize scattered acidic residues to coordinate Mg^2+^, the *trans*-IDS employ two aspartate-rich DDxxD motifs, simply termed the first (FARM) and second (SARM), to bind a trio of Mg^2+^ (Figure 1) [11]. 

The human pathogen *Mycobacterium tuberculosis* encodes an interesting set of IDSs that play various roles in construction of an array of isoprenoids/terpenoids. These serve key roles in construction of the complex mycobacterial cell wall. For example, in production of the polyprenyl phosphate serving as a glyco-carrier required for cell wall biosynthesis [6]. In particular, this 10 isoprenyl-unit carrier is produced via an unusual (*Z,E*)-farnesyl diphosphate (FPP) intermediate, which serves as the initial allylic substrate for the long-chain *cis*-IDS Rv2361c [12]. In turn, the 15-carbon (*Z,E*)-FPP is produced by the short-chain *cis*-IDS Rv1086, which uses (*E*)-geranyl diphosphate (GPP) as its allylic substrate [13]. This unusual bacterial production of the 10-carbon GPP is mediated by the *trans*-IDS Rv0989c (*grcC2*) [14]. 

An intriguing cell wall lipid that appears to be unique to *M. tuberculosis* is the diterpenoid (20-carbon) nucleoside tuberculosinyl-adenosine [15]. This is generated by cyclization of (*E,E,E*)-geranylgeranyl diphosphate (GGPP) to tuberculosinyl/halima-5,13*E*-dienyl diphosphate catalyzed by Rv3377c [16,17], and subsequent addition of adenosine catalyzed by Rv3378c [18]. A role for this diterpenoid in suppressing acidification of the phagosomal compartments into which *M. tuberculosis* are taken up, which assists infiltration of the engulfing macrophage that then serve as host cells, was indicated by a genetic screen [19]. Indeed, the diterpenoid itself appears to suppress phagosomal acidification [20]. However, the genetic screen only identified Rv3377c and Rv3378c as essential for this biosynthetic process [19], and not the *trans*-IDS GGPP synthase Rv3383c (idsB) found in the same operon [21]. This is presumably due to the presence of an additional *trans*-IDS that produces the 20-carbon GGPP in *M. tuberculosis*, Rv0562 (*grcC1*) [21]. 

## 2. Results

**Figure 2:**
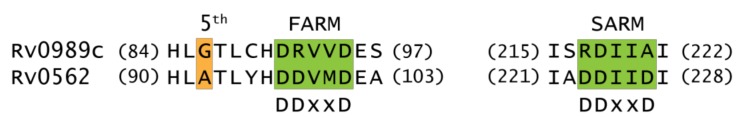
Partial protein sequence alignment of Rv0989c and Rv0562. Rv0562 contains the canonical *trans*-IDS first and second aspartate-rich DDxxD motifs (FARM and SARM; highlighted in green), but these are both disrupted by Arg substitutions in Rv0989c. The fifth residue upstream of the FARM, which is typically responsible for product length determination in *trans*-IDS, is also highlighted here (orange).

As indicated by their shared genetic nomenclature (i.e., *grcC1* and *grcC2*), Rv0562 and Rv0989c are closely related (e.g., particularly among the *M. tuberculosis* IDSs; Figure S1), sharing >56% amino acid sequence identity, despite their divergent product lengths. A common determinant of product chain length in *trans*-IDSs is the identity of the amino acid residue in the fifth position before the FARM [22]. In particular, with smaller residues typically found in GGPP synthases and larger (aromatic) residues in those *trans*-IDS with shorter products. However, Rv0562 and Rv0989 both have small residues at this position (i.e., alanine and glycine, respectively; Figure 2). Thus, there must be an alternative determinant for the difference in product chain length between these two *trans*-IDSs. 

Notably, while Rv0562 contains the canonical DDxxD sequence in both its FARM and SARM, Rv0989c instead contains arginine in place of the second Asp in its FARM and first Asp in its SARM (Figure 2). Although Rv0989c also contains an Ala in place of the last Asp of its SARM, this Asp has been indicated to be less important for *trans*-IDS activity [23]. Indeed, the *M. tuberculosis trans*-IDS that produces (*E,E*)-FPP, Rv3398c (idsA1) [24], has a Gly at this position. By contrast, all the other Asp in these motifs have been shown to be important for catalytic activity [4]. Perhaps not surprisingly then, Arg has not been reported in the FARM or SARM of other *trans*-IDS. Accordingly, it was hypothesized that these unique substitutions in Rv0989c might influence product chain length. This was investigated by iteratively swapping the corresponding active site residues via site-directed mutagenesis between Rv0989c and the closely related Rv0562. 

The resulting mutants were purified and assayed in vitro with DMAPP and IPP. Products were observed during 30-min assays, indicating that these mutants retain reasonable amounts of catalytic activity. However, to ensure thorough representation of all synthesized products, the product ratios reported here are from overnight assays (Table 1). With such extended incubation, wild-type (WT) Rv0989c produces some (*E,E*)-FPP unless IPP concentrations are reduced (Table S1). Thus, the product ratio quantification assays were run with 100 µM DMAPP and 10 µM IPP overnight (Table 1). Under these conditions, WT Rv0989c produces only GPP, and WT Rv0562 still produces only GGPP. By contrast, all of the mutants yielded a variety of products. In general, substitution of Asp for the Arg in Rv0989c led to the production of longer chain product, specifically (*E,E*)-FPP (but not GGPP), in addition to GPP. Conversely, substitution of Arg for the corresponding Asp in Rv0562 led to the appearance of the shorter chain products, both (*E,E*)-FPP and GPP, with only small amounts of GGPP produced. With both of these *M. tuberculosis trans*-IDSs, while the single mutants certainly affect product outcome, the double-mutant led to the greatest change in product outcome i.e., these exhibit additive effects. 

These results indicate that the unique Arg observed in the FARM and SARM of Rv0989c play a role in the unusual production of the 10-carbon GPP by this *M. tuberculosis trans*-IDS. This hypothesis is supported not only by the ability of substituting Asp for these Arg to increase Rv0989c product chain length, but also the inverse decrease in product chain length observed upon substituting Arg for the corresponding Asp in the closely related Rv0562 (Table 1). While the second Asp in the FARM does not typically engage the Mg^2+^ co-factors, the first Asp in the SARM usually does interact with one of this trio of divalent metal ions, specifically Mg^2+^_B_, to which this Asp provides the major enzymatic contact [11]. Thus, it seems likely that this Mg^2+^_B_ is not present in the reactions catalyzed by Rv0989c. However, the Arg found in the SARM potentially replaces this specific co-factor by directly interacting with the diphosphate moiety of the allylic substrate (e.g., much like the binding of the diphosphate moiety of IPP shown in Figure 1). In addition, the Arg found in the FARM presumably shifts this positioning of this motif, bound Mg^2+^ and, consequently, also the allylic substrate. Accordingly, it seems likely that these Arg shift the position of the allylic substrate in a manner that decreases the available space for the appended isoprenyl chain and, hence, limit the size of the final product. Regardless, although determination of the exact mechanism will require more detailed structural analysis, the ability of Arg in the FARM and SARM to affect product chain length, at least in these *M. tuberculosis trans*-IDSs, represents a novel means by which such a final product outcome can be controlled. 

## 3. Materials and Methods

### 3.1. General Reagents

Unless otherwise indicated, reagents were obtained from Sigma-Aldrich (St. Louis, MO, USA). Isoprenoid substrates and standards were purchased from Isoprenoids.com (Tampa, FL, USA). Primers were synthesized by and purchased from Integrated DNA Technologies (Coralville, IA, USA).

### 3.2. Sequence Analysis and Alignment

Protein sequences encoded by *M. tuberculosis* H37Rv loci Rv0562, Rv0989c, Rv1086, Rv2173, Rv2361c, Rv3383c, and Rv3398c were aligned using the MUSCLE algorithm, with subsequent phylogenetic analysis using the maximum likelihood method with the JJT frequencies model with inclusion of a gamma distribution, use of all sites, and 1000 replicates for the bootstrap test, in the MEGA7 software package [25]. 

### 3.3. Site-Directed Mutagenesis

Rv0989c and Rv0562 have previously been cloned, purified, and characterized [14,21]. Site-directed mutagenesis was performed using overlapping mutagenic primers (Figure S2) on pENTR/SD/D-TOPO constructs of each gene. Final constructs were confirmed via complete sequencing prior to transfer to pDEST17 vector for protein expression and purification (Invitrogen, Carlsbad, CA, USA). 

### 3.4. Protein Expression and Assay

Constructs were transformed into *Escherichia coli* BL21-Star (Invitrogen, Carlsbad, CA, USA) or C41 OverExpress cells (Lucigen, Middleton, WI, USA). Starter cultures were inoculated into 10 mL NZY media (10 g/L NaCl, 10 g/L casein, 5 g/L yeast extract, 1 g/L MgSO4 (anhydrous), pH 7.0) with 50 µg/mL carbenicillin and incubated at 200 rpm and 18 °C for 3 days. Starter cultures (5 mL) were used to inoculate 1000 mL fresh NZY media, also with 50 µg/mL carbenicillin, and after reaching an OD_600_ of 0.6 they were induced with 1 Mm Isopropyl β-d-1-thiogalactopyranoside (IPTG) and incubated under continuous shaking at 200 rpm at 18 °C for 12 hours. Cells were harvested by centrifugation at 5000× *g* for 10 min. The cell pellet was re-suspended in 5 mL 25 mM 3-(N-morpholino)-2-hydroxypropanesulfonic acid buffer (MOPSO), pH 7.2, 10 mM MgCl_2_, 10% (*v*/*v*) glycerol with 10 mM imidazole, and homogenized using an EmulsiFlex C-5 (Avestin, Canada). The homogenized suspension was centrifuged at 16,000× *g* for 60 min. The supernatant was passed over 1 mL Ni-NTA agarose (Qiagen, Hilden, Germany) washed with 5 mL buffer containing 10 mM imidazole and an additional 5 mL with 50 mM imidazole. Proteins were eluted with 2 mL buffer containing 250 mM imidazole.

Enzyme assays were carried out with 300 µg purified protein in 2 mL 25 mM MOPSO, pH 7.2, 10 mM MgCl2, 10% (*v*/*v*) glycerol with the addition of 100 µM DMAPP and 10 or 100 µM IPP, the assays were incubated for either 30 minutes or 24 hours at 30 °C before addition of 200 units of calf intestinal alkaline phosphatase (Promega, Madison, WI) and the supplied buffer. The assay was dephosphorylated for 24 hours at 30 °C and extracted three times with 2 mL pentane. The organic phase was combined and concentrated under a gentle stream of N_2_ until a volume of 200 µL solvent remained. The concentrated organic extracts (1 µL) were injected into 3900 Saturn GC (Varian, Palo Alto, CA) with an injector temperature of 250 °C coupled to a Saturn 2100T ion trap mass spectrometer detector for product identification. Separation was achieved using a HP-5MS column (30 m × 250 µm × 0.25 µm) (Agilent, Santa Clara, CA, USA). The chromatographic program was as follows: 50 °C for 3 min, increasing to 300 °C by 15 °C/min and held at 300 °C 3 min. Electrospray ionization scanning from 60–650 *m*/*z* was used to obtain product spectra (Figure S3). Quantification of products was completed using a Shimadzu GC02014 with flame ionization detection (GC-FID) over an SH-Rxi-5ms column (15 m × 250 µm × 0.25 µm; Shimadzu, Kyoto, Japan) using the previously described method. Products were confirmed by comparison, of both retention time and mass spectra (Figure S3), to isoprenoid diphosphate authentic standards dephosphorylated as above.

## Figures and Tables

**Table 1:** Product profiles of Rv0562, Rv0989c, and associated mutants. Assays were completed with purified enzyme in the presence of 100 µM DMAPP and 10 µM IPP for 12 hours prior to dephosphorylation and extraction with organic solvent. Organic extracts were concentrated and analyzed via GC-FID. Product identity was confirmed via comparison to dephosphorylated authentic standards prior to integration of peak area. Product profile is represented as percentage of total isoprenoid peak areas.

Enzyme	GPP	FPP	GGPP
Rv0989c	100	0	0
Rv0989c:R92D	76	24	0
Rv0989c:R217D	40	60	0
Rv0989c:R92D/R217D	30	70	0
Rv0562	0	0	100
Rv0562:D98R	66	34	0
Rv0562:D223R	15	51	34
Rv0562:D98R/D223R	9	90	1

## Supplementary Materials

Supplementary materials are available on line.
