# Supplementary material for: Arginine in the FARM and SARM: A Role in Chain-Length Determination for Arginine in the Aspartate-Rich Motifs of Isoprenyl Diphosphate Synthases from Mycobacterium tuberculosis
Source: Molecules. 2018 Oct 6;23(10):2546. doi: 10.3390/molecules23102546 (PMC6214179; doi:10.3390/molecules23102546)
Supplement: Supplementary file 1 [file molecules-23-02546-s001.pdf]

__Supplementary Data for__:

Arginine in the FARM & SARM: A role in chain-length determination for arginine in the aspartate-rich motifs of isoprenyl diphosphate synthases from *Mycobacterium tuberculosis*

Raimund Nagel,[1] Jill A. Thomas,[1] Faith A. Adekunle,[2] Francis M. Mann,[2,*] Reuben J. Peters[1,*]

Figure S1: Phylogenetic tree of *M. tuberculosis* isoprenyl diphosphate synthases.
Loci number are used to designate each IDS. The phylogenetic tree was constructed in MEGA7 and tested with the Maximum Likelihood algorithm. The JJT with frequencies model was used with inclusion of a gamma distribution. All sites were used and the accuracy of the tree was tested via the bootstrap test with 1000 replicates. The scale bar indicates amino acid changes per site.

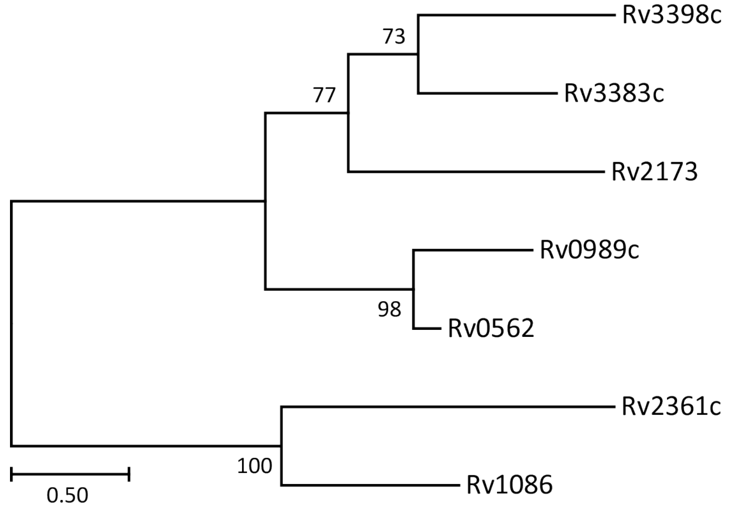

Figure S2: Primer sequences for mutation of Rv0989c and Rv0562

Single mutants were constructed using the primers below. Double mutants were constructed iteratively after confirmation of single mutants via complete sequencing.

Rv0562D98R-F: GCGACCCTCTACCACGATCGCGTGATGGACGAGGCCCAG
Rv0562D98R-R: CTGGGCCTCGTCCATCACGCGATCGTGGTAGAGGGTCGC
Rv0562D223R-F: ACCGCGTTTCAGATCGCCCGGGACATTATCGACATCGAC
Rv0562D223R-R: GTCGATGTCGATAATGTCGCGGGCGATCTGAAACGCGGT
Rv0989cR92D-F: GGACGCTTTGTCACGACGACGTCGTGGATGAGTCCGA
Rv0989cR92D-R: TCGGACTCATCCACGACGTCGTCGTGACAAAGCGTCC
Rv0989cR217D-F: CTGCGTTTGAGATCTCGGACGACATCATCGCCATCTCC
Rv0989cR217D-R: GGAGATGGCGATGATGTCGTCCGAGATCTCAAACGCAG

Figure S3: Mass spectra for enzyme assay products

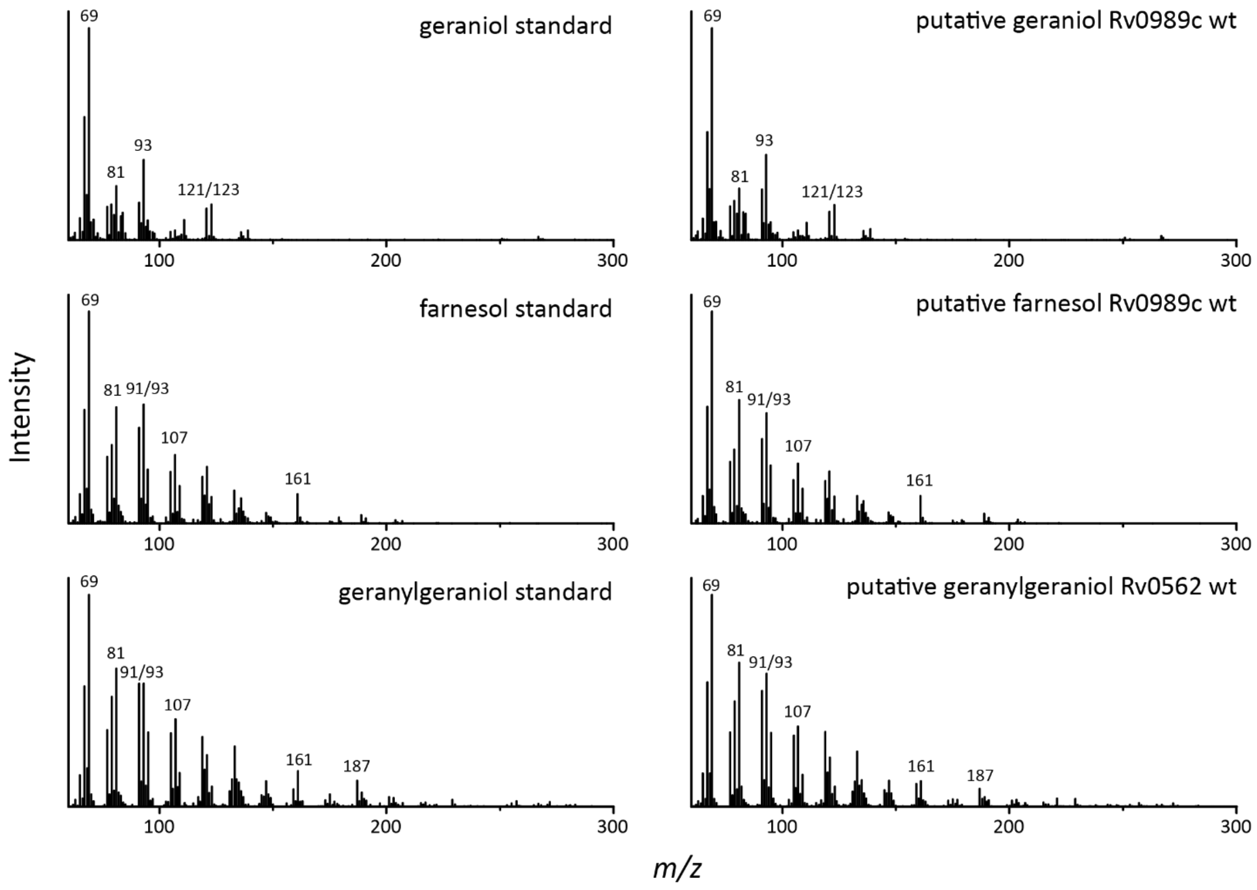

Table S1: Quantification of product profiles of Rv0562, Rv0989c, and associated mutants in the presence of excess IPP

Assays were completed with purified enzyme in the presence of 100 µM DMAPP and 100 µM IPP for 12 hours prior to dephosphorylation and extraction with organic solvent. Organic extracts were concentrated and analyzed via GC-FID. Product identity was confirmed via comparison to dephosphorylated authentic standards prior to integration of peak area.

Enzyme	GPP	FPP	GGPP
**Rv0989c**	55	45	0
**Rv0989c R92D**	78	22	0
**Rv0989c R217D**	60	40	0
**Rv0989c R92/217D**	42	57	0
**Rv0562**	0	0	100
**Rv0562 D98R**	1	2	97
**Rv0562 D223R**	1	9	90
**Rv0562 D98/223R**	14	72	14
